# Evolutionary restoration potential evaluated through the use of a trait‐linked genetic marker

**DOI:** 10.1111/eva.12471

**Published:** 2017-03-27

**Authors:** Travis M. Apgar, Devon E. Pearse, Eric P. Palkovacs

**Affiliations:** ^1^ Department of Ecology and Evolutionary BiologyUniversity of CaliforniaSanta CruzCAUSA; ^2^ Southwest Fisheries Science CenterNational Marine Fisheries ServiceSanta CruzCAUSA

**Keywords:** anadromy, dam removal, ecological restoration, fish passage, freshwater resident, life history variation, *Oncorhynchus mykiss*, rapid evolution

## Abstract

Human‐driven evolution can impact the ecological role and conservation value of impacted populations. Most evolutionary restoration approaches focus on manipulating gene flow, but an alternative approach is to manipulate the selection regime to restore historical or desired trait values. Here we examined the potential utility of this approach to restore anadromous migratory behavior in coastal California steelhead trout (*Oncorhynchus mykiss*) populations. We evaluated the effects of natural and anthropogenic environmental variables on the observed frequency of alleles at a genomic marker tightly associated with migratory behavior across 39 steelhead populations from across California, USA. We then modeled the potential for evolutionary restoration at sites that have been impacted by anthropogenic barriers. We found that complete barriers such as dams are associated with major reductions in the frequency of anadromy‐associated alleles. The removal of dams is therefore expected to restore anadromy significantly. Interestingly, accumulations of large numbers of partial barriers (passable under at least some flow conditions) were also associated with significant reductions in migratory allele frequencies. Restoration involving the removal of partial barriers could be evaluated alongside dam removal and fishway construction as a cost‐effective tool to restore anadromous fish migrations. Results encourage broader consideration of in situ evolution during the development of habitat restoration projects.

## 1 Introduction

There is an increasing recognition that human‐driven evolution can shape the ecological role and conservation value of impacted populations (Hendry, Farrugia, & Kinnison, 2008; Hendry et al., 2011; Palkovacs, Kinnison, Correa, Dalton, & Hendry, 2012; Palumbi, 2001; Stockwell, Hendry, & Kinnison, 2003). This recognition has led to a growing interest in applying evolutionary principles to inform ecological restoration actions (Carroll et al., 2014; Hendry et al., 2011; Smith, Kinnison, Strauss, Fuller, & Carroll, 2014). In some cases, human activity shifts traits such that important ecological functions are altered or lost (Audzijonyte, Kuparinen, & Fulton, 2014; Audzijonyte, Kuparinen, Gorton, & Fulton, 2013; Palkovacs, Wasserman, & Kinnison, 2011). In such situations, evolutionary strategies can be applied to achieve ecological restoration. Calls to apply evolutionary restoration techniques have largely focused on managing gene flow to increase fitness in threatened populations (Aitken & Whitlock, 2013; Frankham, 2015; Leger, 2013; Whiteley, Fitzpatrick, Funk, & Tallmon, 2015). A somewhat different approach that has received less attention is to estimate the effects of anthropogenic impacts on key traits and then to manipulate selection regimes in ways that restore trait values. In this study, we develop an approach to restoration planning that considers predicted evolutionary responses to potential habitat restoration actions.

In many ecosystems, humans have altered selection regimes either directly through selective mortality (e.g., commercial fisheries, trophy hunting) or indirectly through habitat modification (e.g., habitat fragmentation, habitat alteration; Palkovacs et al., 2012; Carroll et al., 2014; Smith et al., 2014). For example, fisheries‐induced mortality of anadromous sockeye salmon (*Oncorhynchus nerka*) and alewife (*Alosa pseudoharengus*) appear to have driven changes in life history traits and body size (Davis & Schultz, 2009; Kendall, Dieckmann, Heino, Punt, & Quinn, 2014). Reduced body size translates into a reduction in marine‐derived nutrients brought into freshwater ecosystems, potentially impacting the ecology of stream and riparian habitats (Carlson, Quinn, & Hendry, 2011; Schindler et al., 2003; Twining, Palkovacs, Friedman, Hasselman, & Post, 2016; West, Walters, Gephard, & Post, 2010). In such scenarios, evolutionary restoration via reduced harvest rates and reduced size‐selectivity could help restore both trait values and ecological functions (Dunlop, Eikeset, & Stenseth, 2015; Dunlop, Enberg, Jorgensen, & Heino, 2009). A specific scenario where traits have been altered due to human habitat disturbance is dam construction. Dams fragment rivers and change upstream and downstream habitat, driving changes in selection that can reshape migratory behavior and morphology for impacted fish populations (Haas, Blum, & Heins, 2010; Palkovacs, Dion, Post, & Caccone, 2008). Such trait changes can alter important ecological processes such as food web interactions and nutrient transport (Jones, Palkovacs, & Post, 2013; Palkovacs & Post, 2009; Post, Palkovacs, Schielke, & Dodson, 2008).

Here we apply an evolutionary restoration framework to inform the recovery of coastal California steelhead trout (*Oncorhynchus mykiss*; Walbaum). Steelhead trout display variability in migratory behavior. Both within and among populations, some individuals are anadromous, spawning in freshwater and migrating to the ocean, whereas others are residents, completing their entire life cycle in freshwater (Kendall et al., 2015; Sogard et al., 2012). Populations can rapidly evolve freshwater residency when dams or other barriers impede migratory corridors (Pearse, Miller, Abadía‐Cardoso, & Garza, 2014). Across a broad suite of species, the loss of anadromy has important implications for ecosystems. Anadromous fishes play a critical role in coastal watersheds by connecting ecosystems, driving nutrient dynamics, impacting food web interactions, shaping local species diversity (Flecker et al., 2010; Hocking & Reynolds, 2011; Naiman, Bilby, Schindler, & Helfield, 2002; Schindler et al., 2003; Willson & Halupka, 1995). This ecological role is fundamentally altered when human disturbance, often in the form of dam construction, causes populations to evolve freshwater residency (Palkovacs & Post, 2009; Post et al., 2008).

Anadromous populations of many species have declined substantially over recent decades (Chaput, Cass, Grant, Huang, & Veinott, 2013; Limburg & Waldman, 2009; Rand, Berejikian, Pearsons, & Noakes, 2012). In California, anadromous steelhead populations are at risk of disappearing (Katz, Moyle, Quiñones, Israel, & Purdy, 2013). Extirpation threatens some populations; however, the evolutionary loss of the anadromous life history is a more widespread phenomenon where populations persist but as nonanadromous freshwater residents. Currently, some anadromous steelhead populations in California are listed as either threatened (north of Point Conception, California, USA, to the Klamath River basin) or endangered (south of Point Conception) under the US Endangered Species Act. In contrast, freshwater resident populations, commonly referred to as rainbow trout, are not protected, even though many populations are native and have lost anadromy due to human habitat alteration (Clemento, Anderson, Boughton, Girman, & Garza, 2009). In an ironic twist of fate, freshwater resident rainbow trout has become the most widely distributed freshwater fish in the world due to human introductions, and these invasive rainbow trout originate largely from California hatchery stocks (Crawford & Muir, 2008; Halverson, 2008; Stanković, Crivelli, & Snoj, 2015).

Across a wide variety of fish species, anadromy and freshwater residency evolve rapidly, although individual decisions to migrate or remain resident depend on interactions among genetic, individual condition, and environmental factors (Dodson, Aubin‐Horth, Thériault, & Páez, 2013; Hendry, Bohlin, Jonsson, & Berg, 2004). Anadromy may benefit some individuals by allowing them to escape stressful conditions in freshwater (i.e., reduced food supply, harmful flows, etc.) and providing opportunities for increased growth in the ocean and ultimately higher fecundity (Hendry et al., 2004). In California, females comprise a larger proportion of anadromous individuals in some populations, presumably because of the fitness benefits of greater fecundity for females (Ohms et al., 2014; Rundio, Williams, Pearse, & Lindley, 2012; Satterthwaite et al., 2010). But anadromy is costly during the migratory period and may subject individuals to increased energy expenditures and elevated risks of mortality through physiological stress and predation. Theory therefore predicts that anadromy should become less favored as freshwater growth rate increases (or marine productivity decreases), and if the risk of migrating to the ocean increases mortality (Hendry et al., 2004).

Because of widespread variation in migratory behavior within and among populations of steelhead trout, the determinants of anadromy and residency in this species have received much attention (Berejikian, Bush, & Campbell, 2014; Hale, Thrower, Berntson, Miller, & Nichols, 2013; Hayes et al., 2012; Kendall et al., 2015; Pearse et al., 2014; Phillis et al., 2016; Satterthwaite et al., 2009, 2010; Sloat & Reeves, 2014). Quantifying the proportion of anadromous steelhead *vs*. resident rainbow trout in a population typically requires directly observing the behavior of a large number of individuals. However, the distribution of adaptive genomic variation associated with specific traits has the potential to provide inference about the selective environments and adaptive difference among populations.

In coastal California watersheds, a region of *O. mykiss* chromosome 5 (*Omy5*) has been recently identified, the Omy5 migration‐associated region (MAR), with alternate alleles being tightly associated with the population prevalence of either migration or freshwater residency (Leitwein, Garza, & Pearse, 2017; Pearse et al., 2014). Many loci in the MAR are in strong linkage disequilibrium, suggesting the presence of a chromosomal inversion with loci associated with anadromous migratory traits (Leitwein et al., 2017; Pearse et al., 2014). Some of these traits include smoltification, growth rate, survival in sea water, and observed out‐migration of juveniles (Doctor, Berejikian, Hard, & Vandoornik, 2014; Hecht, Hard, Thrower, & Nichols, 2015; Pearse et al., 2014; Phillis et al., 2016). In one example, a population recently translocated from below to above a waterfall has undergone a 49% reduction in the frequency of anadromy‐associated alleles, a 19% reduction in smoltification, a 37% decrease in survival when exposed to sea water, and a 25% reduction in observed juvenile out‐migration (Pearse et al., 2014; Phillis et al., 2016). Thus, although a single genomic locus should not be considered representative of all the adaptive genomic variation associated with this complex phenotype, variation in MAR allele frequencies does provide substantial utility for evaluating evolutionary restoration as a conservation tool (Pearse, 2016). Here we evaluated the effects of natural and anthropogenic environmental variables on the observed frequency of MAR alleles across 39 steelhead trout populations and modeled the potential for evolutionary restoration of anadromy at sites that have been impacted by anthropogenic barriers.

## 2 Materials and Methods

### 2.1 Modeling overview

The overall goal of our modeling exercise was to link environmental variables such as climate, geomorphology, and migratory barriers to the frequency of MAR alleles associated with anadromy in steelhead populations across California. We then used model predictions to inform conservation strategies aimed at restoring anadromous migratory behavior to populations that have lost anadromy due to human habitat modification.

### 2.2 Sample collection and genotyping

**Figure 1:**
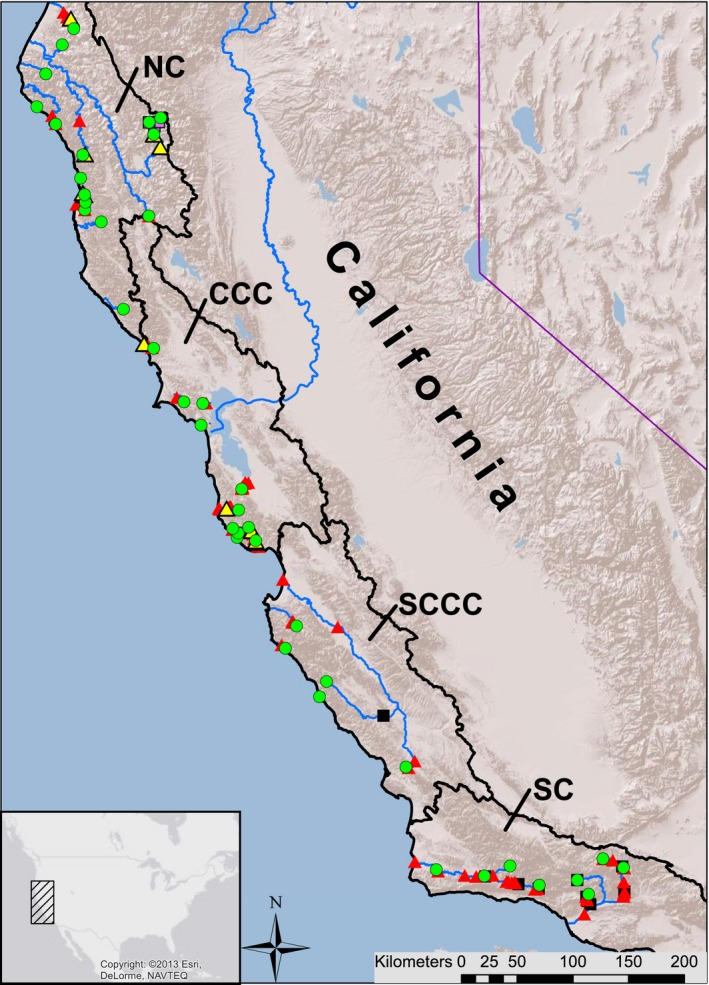
California *Oncorhynchus mykiss* sampling locations with different barrier types georeferenced along the migration path. The Distinct Population Segments from North to South are as follows: Northern California (NC), Central California Coast (CCC), South‐Central California Coast (SCCC), and Southern California (SC). *Sampling Locations* are represented by hollow/green circles, and their corresponding *Sampling Streams* are solid/blue lines. *Partial Natural Barriers* are represented by yellow/hollow triangles, while *Partial Anthropogenic Barriers* are solid/red triangles. *Complete Natural Barriers* are hollow/purple squares, and *Complete Anthropogenic Barriers* are solid/black squares

Genetic samples were collected from coastal California steelhead populations as part of earlier studies to assess population genetic structure within and among distinct population segments (DPSs; Clemento et al., 2009; Garza et al., 2014; Pearse et al., 2014). We examined 1,332 samples from 39 populations collected in 2001. Populations sampled belong to four DPSs: Southern California (SC), South‐Central California Coast (SCCC), Central California Coast (CCC), and Northern California (NC; Figure 1). Single nucleotide polymorphisms were genotyped following Pearse and Garza (2015), including two loci linked to the chromosome Omy5 MAR (Abadía‐Cardoso et al., 2016; Leitwein et al., 2017; Pearse et al., 2014). The alternative alleles at loci in this region show strong differences in frequency between predominantly anadromous versus predominately resident populations (Abadía‐Cardoso et al., 2016; Leitwein et al., 2017; Pearse & Garza, 2015; Pearse et al., 2014). For convenience, we hereafter refer to these as “anadromous” and “resident” alleles. The frequency of haplotypes associated with anadromy, **ƒ(A),** was calculated as the sum of the anadromy‐associated alleles over the total number of alleles in the population at the locus Omy114448 (Abadía‐Cardoso, Clemento, & Garza, 2011; Pearse et al., 2014).

### 2.3 Environmental variables

A range of environmental variables shape the contemporary evolution of anadromous migratory behavior in fishes (Table 1; Hendry et al., 2004; Quinn, 2005; Quinn & Myers, 2005). Climatological variables associated with anadromy include rainfall, runoff, streamflow, and baseflow. Geomorphological variables include streambed geology, stream order, stream gradient, riparian vegetation, elevation, stream temperature, maximum air temperature, and migration distance. Natural and anthropogenic barriers to instream migration fall into two broad categories. Partial barriers impede but do not entirely prevent riverine migration. These barriers are surmountable under most flow conditions; however, they do impart an energetic cost to migration (Jonsson, Castro‐Santos, & Letcher, 2010). Complete barriers block upstream migration entirely, but opportunities for downstream migration are possible if a fish can survive passage over a waterfall or dam, and in some cases through hydroelectric turbines. Both partial and complete barriers may be either natural features (e.g., waterfalls, rapids, sandbars, log jams) or anthropogenic disturbances (e.g., road crossing, culverts, water diversions, dams). While all anthropogenic disturbances are relatively recent, natural landscape features may isolate populations for long periods of time (e.g., large waterfalls), while others may only be temporary (e.g., log jams).

**Table 1:** Environmental variables included in the model based on possible effects on anadromy

Environmental conditions affecting migration			
Climatological	In‐stream Barriers	Geomorphology	
Runoff	Partial Anthropogenic Barriers	Streambed Geology	Migration Distance
Rainfall	Complete Anthropogenic Barriers	Stream Order	Elevation
Streamflow	Partial Natural Barriers	Stream Gradient	Stream Temp
Baseflow	Complete Natural Barriers	Riparian Vegetation	Max Air Temp

Using ArcGIS 10.2 (ESRI 2015), we created point shapefiles for each georeferenced sampling location. We then constructed polyline shapefiles from each respective sampling point to the ocean to represent the stream‐path, which was used to calculate migration distance for the freshwater portion of the migration. GIS layers for climatological and geomorphological variables were downloaded on December 13, 2013 via the OSU Prism (PRISM Climate Group, Oregon State University, http://prism.oregonstate.edu), Geospatial Gateway (USDA, https://gdg.sc.egov.usda.gov/), and CalAtlas (CNRA, http://www.atlas.ca.gov/download.html) databases. The California Fish Passage Assessment Database (CFPAD; www.calfish.org/tabid/420/Default.aspx) was used to identify all the potential barriers to migratory fish along each stream‐path. Based on barriers cataloged in this database, we calculated the number of barriers within each category occurring along each migratory pathway that were present prior to genetic sampling in 2001 (see Supplementary Methods 1). We classified barriers as partial or complete and as natural or anthropogenic (Table 1). Partial barriers are those in‐stream barriers that are considered passable in an upstream direction by anadromous fishes under at least some flow conditions. Complete barriers are insurmountable in an upstream direction under all flow conditions. The effects of partial barriers were considered to be additive, as they can consecutively impart an energetic cost along the migration path (Jonsson et al., 2010). In contrast, the effects of complete barriers were considered to be binary (present/absent), as they function to block all upstream movement.

### 2.4 Statistical framework

In order to determine which environmental variables contributed significantly to variation in the frequency of anadromy‐associated alleles, we conducted backward stepwise regressions for model selection to establish Akaike's Information Criterion (AIC) for each combination of variables. The minimum AIC value was used to select a best‐fit model. The frequency of the anadromous allele **ƒ(A)**, at each sampling location was used as the dependent variable. Relative effect contributions for each factor were estimated as the amount of change in the population's haplotype frequency when a given factor was included or excluded from the model. Model validation was conducted in two ways. First, observed **ƒ(A)** was plotted against predicted **ƒ(A)** using a simple liner regression. Predicted **ƒ(A)** values were calculated using the best‐fit model. We used an *R* ^2^ value and 95% prediction interval to evaluate model accuracy. The 95% prediction interval accounts for the uncertainty of predicting a single observation in the model when compared to the 95% confidence interval, which is used to evaluate the mean values of the dataset. Second, bootstrap values were generated by taking 10,000 iterations of subsamples of the independent variables and using a *p*‐value of <.05. We then quantified the proportions of times the term was below the *p*‐value significance threshold and reported it as frequency of when the term was included in the model. Analyses were performed in JMP Pro 12 (SAS 2015).

### 2.5 Evolutionary restoration

Using the relative effect contributions determined by the best‐fit model, we calculated the expected evolutionary responses (predicted **ƒ(A)**) for each population in a scenario where all anthropogenic barriers were removed from the downstream watershed. We then considered the change in the frequency of the anadromous allele **ΔA** under current versus restored scenarios as our measure of potential for evolutionary restoration. Then we assessed the potential for evolutionary restoration for each DPS, as these are the primary regional management units for coastal California.

When accurate cost estimates are available, our evolutionary restoration framework can be used to inform management of which watersheds to restore and which specific barriers to remove. This approach allowed us to compare the theoretical effectiveness of various barrier removal scenarios and to determine what types of barriers and which watersheds can yield the greatest evolutionary restoration at the lowest dollar cost. We obtained cost estimates for specific barrier types and watersheds within our study range from the Pacific States Marine Fisheries Commission (PSMFC). The PSMFC has been compiling cost estimates with the goal of incorporating them into their Passage Assessment Database, which contains all the potential barriers to anadromy along the Pacific coast. We were able to use exact cost estimates or approximate removal costs based on barrier type for Lion Canyon Creek, South Fork Bear Creek, Santa Paula Creek, Los Trancos Creek, Boulder Creek, and the Nacimiento River (PSMFC, unpublished data).

## 3 Results

The AIC best‐fit model contained five terms explaining significant variation in **ƒ(A**; Table 2). Migration distance was the sole climatological or geomorphological variable selected in the model. The largest effect contributions were due to the presence of complete anthropogenic or natural barriers. Complete natural barriers had the highest effects contribution at ±30.66% with a bootstrap frequency of 0.95. Complete anthropogenic barriers had an effect contribution of ±18.47% with a bootstrap value of 0.93. The remaining three terms had an additive effect in the model and thus had negative effect contributions. Partial anthropogenic barriers had an effect contribution of −1.82% per barrier and a bootstrap value of 0.55. Migration distance had a −6.79% per 100 km and a bootstrap value of 0.53. Finally, partial natural barrier effect contribution was calculated to be −0.51% per barrier along the migration path with a bootstrap value of 0.08. Even though this term was not significant, its presence helped increase the overall accuracy of the model.

**Table 2:** Model output representing relative effect contributions and bootstrapping results for population haplotype frequencies. Complete natural and anthropogenic barriers are presence (−) absence (+) terms, while partial natural and anthropogenic barriers and migration distance have additive effects

Effect contribution results				Bootstrapping results	
Environmental variable	Effect contribution	Standard error	Units	*N* (10,000) *p* < .05	Frequency in model
Complete natural barriers	±30.66%	0.0540	Yes = negative, No = positive	9,488	0.95
Complete anthropogenic barriers	±18.47%	0.0400	Yes = negative, No = positive	9,343	0.93
Partial anthropogenic barriers	−1.82%	0.0079	* number of barriers	5,523	0.55
Migration distance	−6.79%	0.0329	per 100 km	5,288	0.53
Partial natural barriers	−0.51%	0.0319	* number of barriers	797	0.08

**Figure 2:**
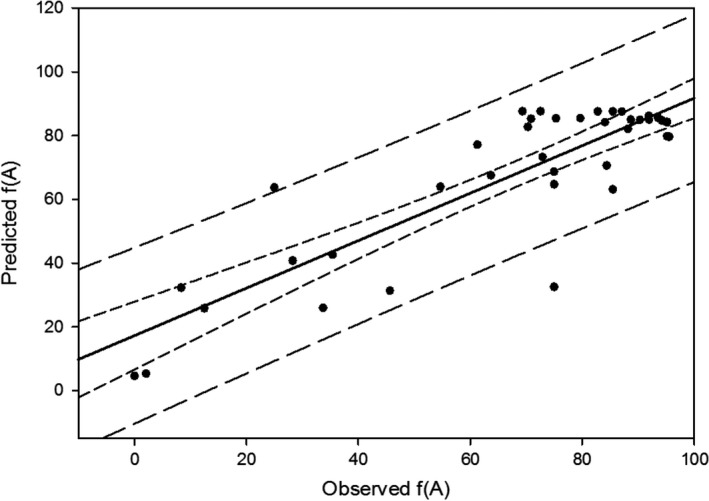
Observed versus predicted anadromous allele frequency **ƒ(A)** for each sampling location using the best‐fit model predictions. Short‐dashed line represents 95% confidence interval; long‐dashed line represents 95% prediction interval. Two outlier points are Tassajera Creek and Nacimiento River from the Salinas River watershed. Tassajera Creek is at the head of a highly agricultural watershed that experiences main stem seasonal drying from agricultural withdrawals. The Nacimiento River population may exhibit adfluvial migrations downstream into Nacimiento Lake

Model validation through linear regression of observed versus predicted **ƒ(A)** had all but two data points falling within the 95% prediction interval (*R* ^2^ = 0.745; Figure 2). The two sampling sites falling outside of the prediction interval were both within the Salinas River watershed, which is a large river system with diverse habitats. Historical or contemporary factors may be present in this drainage that caused our model to perform poorly. The model performed well for all other sampling sites.

**Figure 3:**
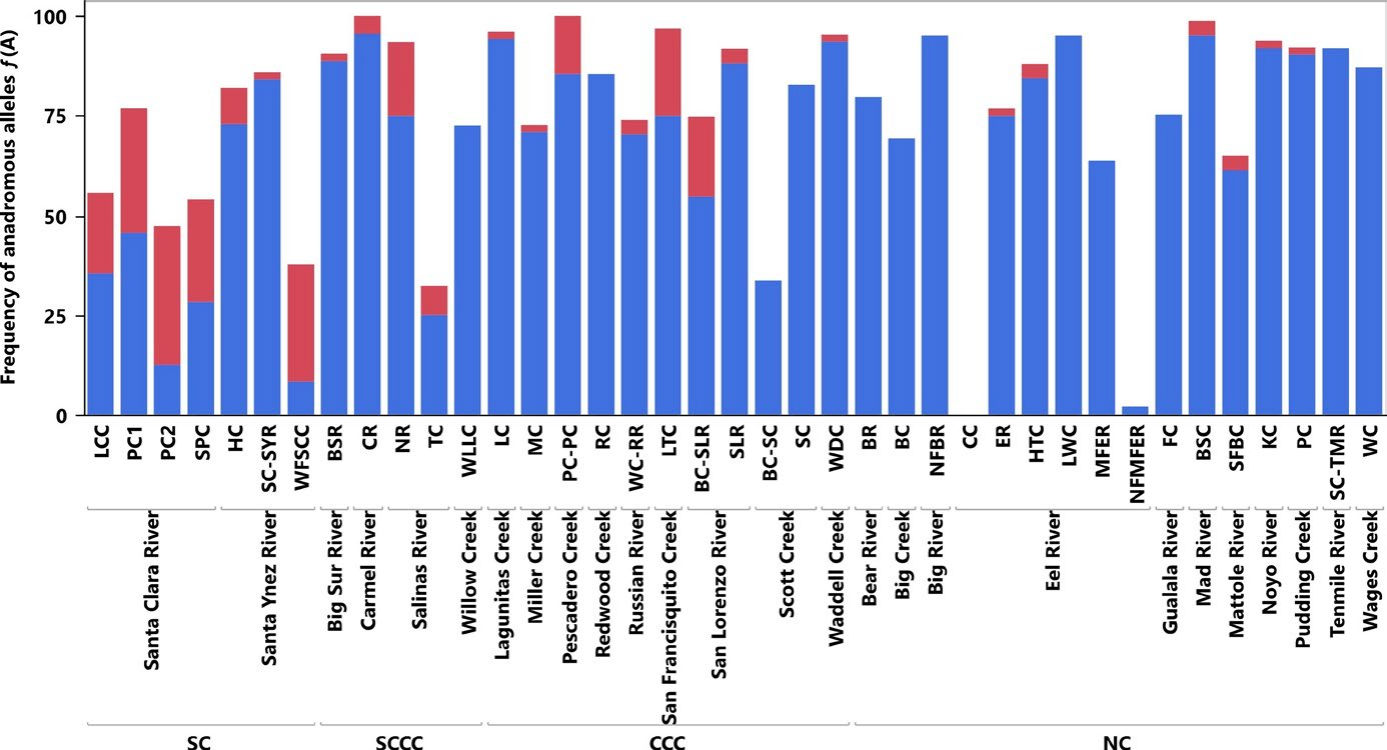
Observed versus restored anadromous allele frequency **ƒ(A)** for each sampling location grouped by watershed and DPS. Blue bars represent measured **ƒ(A)**, red bars represent restored **ƒ(A)**. Restored **ƒ(A)** is the calculated increase in anadromy‐associated alleles predicted if all anthropogenic barriers were removed, thus showing overall restoration potential for each location. Site abbreviations follow those given in Table 4

Frequency of anadromy **ƒ(A)** generally decreased north to south, while restoration potential **ΔA** generally increased from north to south (Figure 3, Table 3). However, considerable variation among sampling locations was found in every DPS (Table 4). The *Southern California* (SC) DPS is within a highly urbanized and anthropogenically impacted region (Fig. S1). The average **ƒ(A)** in SC was 41.02%, the lowest of any DPS. It also had the highest average number of partial anthropogenic barriers per watershed (*n* = 4.7, range 1–9) and the highest total number of complete anthropogenic barriers (*n* = 8). The SC also had a relatively long average potential migration distance at 80.46 km. Rugged coastal mountains and agricultural land dominate the *South‐Central California Coast* (SCCC) DPS (Fig. S2). The average **ƒ(A)** in SCCC was 71.37%. The average number of partial anthropogenic barriers was 1.6 per watershed (range 0–4), and there was only one complete anthropogenic barrier affecting our sampled populations in this DPS. The SCCC had the longest average migration distance at 112.26 km due to the inclusion of the Salinas River. The *Central California Coast* (CCC) DPS is a mix of rugged coast and urbanized areas (San Francisco Bay Area; Fig. S3). The average **ƒ(A)** was 75.84%, which was the highest of any DPS evaluated. The average number of partial anthropogenic barriers was 3.8 per watershed (range 0–12), and there were no complete anthropogenic barriers present but one complete natural barrier affecting our sampled populations. The average migration distance was shortest of all the DPS's at just 12.91 km. The *Northern California* (NC) DPS is the least urbanized section of coastal California, although impacts from forestry and illegal marijuana cultivation (Bauer et al., 2015) are widespread (Fig. S4). The average **ƒ(A)** was 72.35%, which was the second highest of all the DPS's. The average number of partial anthropogenic barriers was 0.5 per watershed (range 0–2), the lowest of any DPS considered. There were no complete anthropogenic barriers in this DPS but two complete natural barriers affecting our sampled populations. The average migration distance of NC was 103.31 km, the second highest of any DPS considered.

**Table 3:** Summary of anadromous allele frequency **ƒ(A)** by Distinct Population Segment (DPS). The Southern California DPS contains the greatest amount of anthropogenic disturbance across all our sampling locations in the form of partial and complete anthropogenic barriers. This is reflected in the average restoration potential **ΔA**, which is much higher than the other DPSs

DPS	Southern California (SC)	South‐Central California Coast (SCCC)	Central California Coast (CCC)	Northern California (NC)
Average f(A)	41.02	71.37	75.84	72.35
Average ΔA	21.77	6.40	6.28	1.02
Status	Endangered	Threatened	Threatened	Threatened
Average Partial Anthro Bar No.	4.7	1.6	3.8	0.5
Average Partial Natural Bar No.	0	0	0.64	0.81
Total Complete Anthro Bar No.	8	1	0	0
Total Complete Natural Bar No.	0	0	1	3
Average Migration Distance	80.46	112.26	12.91	103.31

**Table 4:** Individual sampling locations and their corresponding data sorted from south to north and organized by watershed

Sampling Location	Location Codes	Latitude	Longitude	Major Watershed	DPS	Status	ƒ(A)	Restored ƒ(A)	ΔA	Partial Natural Bar No.	Complete Natural Bar PA	Partial Anthro Bar No.	Complete Anthro Bar PA	Migration Distance (km)
Santa Paula Creek^b^^,^ c	SPC	34.444837	−119.068914	Santa Clara River	SC	Endangered	**28.26**	**54.01**	**25.75**	0	No	4	Yes	39.42
Lion Canyon Creek^b^^,^ c	LCC	34.549924	−119.16586	Santa Clara River	SC	Endangered	**35.42**	**55.71**	**20.29**	0	No	1	Yes	91.9
Piru Creek^b^^,^ c	PC1	34.635302	−118.756754	Santa Clara River	SC	Endangered	**45.65**	**76.86**	**31.21**	0	No	7	Yes	96.91
Piru Creek^b^^,^ c	PC2	34.703043	−118.937168	Santa Clara River	SC	Endangered	**12.5**	**47.35**	**34.85**	0	No	9	Yes	124.33
Hilton Creek^b^^,^ c	HC	34.586241	−119.986434	Santa Ynez River	SC	Endangered	**72.92**	**82.02**	**9.1**	0	No	5	No	76.85
Salsipuedes Creek^b^^,^ c	SC‐SYR	34.633739	−120.412621	Santa Ynez River	SC	Endangered	**84.09**	**85.91**	**1.82**	0	No	1	No	24
West Fork Santa Cruz Creek^b^^,^ c	WFSCC	34.657939	−119.759101	Santa Ynez River	SC	Endangered	**8.33**	**37.72**	**29.39**	0	No	6	Yes	109.81
Willow Creek^c^	WLLC	35.893746	−121.460507	Willow Creek	SCCC	Threatened	**72.58**	**72.58**	**0**	0	No	0	No	0.13
Big Sur River^c^	BSR	36.245949	−121.773275	Big Sur River	SCCC	Threatened	**88.71**	**90.53**	**1.82**	0	No	1	No	12.11
Carmel River^a^, ^b^^,^ c	CR	36.409742	−121.674336	Carmel River	SCCC	Threatened	**95.57**	**100**	**4.43**	0	No	3	No	37.55
Tassajera Creek^b^	TC	35.38441	−120.682174	Salinas River	SCCC	Threatened	**25**	**32.28**	**7.28**	0	No	4	No	243.73
Nacimiento River^b^	NR	36.00643	−121.398949	Salinas River	SCCC	Threatened	**75**	**93.47**	**18.47**	0	No	0	Yes	267.82
San Lorenzo River^b^	SLR	37.029971	−122.057524	San Lorenzo River	CCC	Threatened	**88.18**	**91.82**	**3.64**	2	No	2	No	12.29
Boulder Creek^e^	BC‐SLR	37.126409	−122.123467	San Lorenzo River	CCC	Threatened	**54.69**	**74.71**	**20.02**	3	No	11	No	29.77
Scott Creek^b^	SC	37.050498	−122.226909	Scott Creek	CCC	Threatened	**82.78**	**82.78**	**0**	0	No	0	No	1.45
Big Creek^b^	BC‐SC	37.083094	−122.217591	Scott Creek	CCC	Threatened	**33.7**	**33.7**	**0**	0	Yes	0	No	5.89
Waddell Creek^c^	WDC	37.116208	−122.268818	Waddell Creek	CCC	Threatened	**93.55**	**95.37**	**1.82**	0	No	1	No	3.65
Peters Creek^a^, ^e^	PC‐PC	37.251634	−122.218089	Pescadero Creek	CCC	Threatened	**85.48**	**100**	**14.52**	1	No	12	No	30.46
Los Trancos Creek^c^	LTC	37.405982	−122.193483	San Francisquito Creek	CCC	Threatened	**75**	**96.84**	**21.84**	0	No	12	No	14.33
Redwood Creek^d^	RC	37.866403	−122.578553	Redwood Creek	CCC	Threatened	**85.48**	**85.48**	**0**	0	No	0	No	1.23
Miller Creek^d^	MC	38.025405	−122.567561	Miller Creek	CCC	Threatened	**70.88**	**72.7**	**1.82**	0	No	1	No	7.82
Lagunitas Creek^e^	LC	38.034194	−122.743381	Lagunitas Creek	CCC	Threatened	**94.26**	**96.08**	**1.82**	0	No	1	No	16.28
Willow Creek^e^	WC‐RR	38.42017	−123.036371	Russian River	CCC	Threatened	**70.31**	**73.95**	**3.64**	1	No	2	No	11.02
Fuller Creek^c^	FC	38.699424	−123.327231	Gualala River	NC	Threatened	**75.33**	**75.33**	**0**	0	No	0	No	33.12
North Fork Big River^e^	NFBR	39.333469	−123.560718	Big River	NC	Threatened	**95.16**	**95.16**	**0**	0	No	0	No	49.52
Kass Creek^d^	KC	39.417581	−123.719985	Noyo River	NC	Threatened	**91.94**	**93.76**	**1.82**	0	No	1	No	12.01
Pudding Creek^e^	PC	39.472249	−123.716991	Pudding Creek	NC	Threatened	**90.32**	**92.14**	**1.82**	0	No	1	No	13.25
Smith Creek^e^	SC‐TMR	39.527682	−123.728451	Tenmile River	NC	Threatened	**91.94**	**91.94**	**0**	2	No	0	No	6.23
Wages Creek^e^	WC	39.647917	−123.770039	Wages Creek	NC	Threatened	**87.1**	**87.1**	**0**	0	No	0	No	1.88
Big Creek^e^	BC	40.157456	−124.210424	Big Creek	NC	Threatened	**69.35**	**69.35**	**0**	0	No	0	No	0.14
South Fork Bear Creek^c^	SFBC	40.035764	−124.025037	Mattole River	NC	Threatened	**61.29**	**64.93**	**3.64**	0	No	2	No	101.14
Bear River^e^	BR	40.399481	−124.137965	Bear River	NC	Threatened	**79.69**	**79.69**	**0**	0	No	0	No	32.11
Eel River^b^	ER	39.38652	−123.116409	Eel River	NC	Threatened	**75**	**76.82**	**1.82**	0	No	1	No	252.19
Hollow Tree Creek^d^	HTC	39.817585	−123.757815	Eel River	NC	Threatened	**84.38**	**88.02**	**3.64**	1	No	2	No	189.28
Middle Fork Eel River^b^	MFER	39.984042	−123.090546	Eel River	NC	Threatened	**63.72**	**63.72**	**0**	3	No	0	No	272.87
North Fork Middle Fork Eel River^b^	NFMFER	40.072531	−123.13593	Eel River	NC	Threatened	**2.08**	**2.08**	**0**	3	Yes	0	No	286.28
Cutfinger Creek^b^	CC	40.106932	−123.028493	Eel River	NC	Threatened	**0**	**0**	**0**	3	Yes	0	No	296.85
Lawrence Creek^d^	LWC	40.616988	−123.990458	Eel River	NC	Threatened	**95.16**	**95.16**	**0**	0	No	0	No	52.1
Blue Slide Creek^d^	BSC	40.737754	−123.885432	Mad River	NC	Threatened	**95.16**	**98.8**	**3.64**	1	No	2	No	53.99

**f(A)**, frequency of anadromy‐associated alleles; **Restored f(A)**, predicted frequency of anadromy‐associated alleles following restoration; ΔA, restoration potential; **Partial Natural Bar No.**, number of partial natural barriers downstream of sampling location; **Complete Natural Bar PA**, complete natural barrier present (Yes) or absent (No); **Partial Anthro Bar No.**, number of partial anthropogenic barriers downstream of sampling location; **Complete Anthro Bar PA**, complete anthropogenic barrier present (Yes) or absent (No).

a Locations with predicted restored ƒ(A) values >1.0 and capped at 100%.

b Genetic data from Pearse and Garza (2015).

c Genetic data from Abadía‐Cardoso et al. (2016).

d Genetic data from Pearse et al. (2014).

e New data.

Based on the model output, when complete anthropogenic barriers were present, populations had a difference of ±18.47% in anadromy‐associated alleles. However, partial anthropogenic barriers (−1.82% per barrier) can have an additive effect that can equal that of complete barriers (e.g., Los Trancos Creek). Highly urbanized areas have the highest densities of partial and complete anthropogenic barriers, and thus, their restoration potential is higher.

Using the subset of watersheds where there is reliable cost information, we evaluated a few case studies (Table 5). Los Trancos Creek (CCC, Fig. S3) has 12 partial anthropogenic barriers. We estimated the cost to remove all 12 barriers as $2,036,000. The estimated evolutionary response is a 21.84% increase in anadromy for this watershed, representing $93,223 per one percent increase in anadromy. In contrast, some watersheds have large complete anthropogenic barriers such as the 64 m earthen dam on the Nacimiento River (SCCC, Fig. S2). It would cost an estimated $75,000,000 to remove this dam (PSMFC, unpublished data). The estimated evolutionary response in this case is a 18.47% increase in anadromy, which represents a substantially more costly $4,060,638 per one percent increase in anadromy. However, there are some highly urbanized watersheds that have complete anthropogenic barriers that are not large dams but improperly designed culverts or grade structures. These types of barriers prevent upstream movement just as large dams but are considerably less expensive to remove. For example, our model estimated that a 20.29% increase in anadromy‐associated alleles would result from removing a partial barrier and a diversion dam in Lion Canyon Creek (SC, Fig. S1), for only $320,000. This restoration project is estimated to cost $17,551 per one percent increase in anadromy, the highest return on investment for any of the watersheds considered.

**Table 5:** Sampling locations with reliable cost information were used to create a ranking function to generate best return on investment for potential restoration projects. Restored **ƒ(A)** values and remediation costs represent a scenario where all anthropogenic barriers are removed from the migration path

Sampling location	Location code	DPS	Status	ƒ(A)	Restored ƒ (A)	ΔA	Remediation cost	Cost per 1% ƒ(A)	Number of barriers removed	Complete anthro bar removed?
Lion Canyon Creek	LCC	SC	Endangered	35.42	55.71	20.29	$320,000	$15,771	2	**Yes**
South Fork Bear Creek	SFBC	NC	Threatened	61.29	64.93	3.64	$380,000	$104,395	2	No
Santa Paula Creek	SPC	SC	Endangered	28.26	54.01	25.75	$640,000	$24,854	4	**Yes**
Los Trancos Creek	LTC	CCC	Threatened	75.00	96.84	21.84	$2,036,000	$93,223	12	No
Boulder Creek	BC‐SLR	CCC	Threatened	54.69	74.71	20.02	$2,046,400	$102,217	11	No
Nacimiento River	NR	SCCC	Threatened	75.00	93.47	18.47	$75,000,000	$4,060,638	1	**Yes**

**f(A)**, frequency of anadromy‐associated alleles; **Restored f(A)**, predicted frequency of anadromy‐associated alleles following restoration; ΔA, restoration potential;

**Complete anthro bar removed?**, does project include the removal of a complete anthropogenic barrier? (Yes /No).

## 4 Discussion

Ecologists and evolutionary biologists have become increasingly aware that human‐driven evolution can shape key traits of ecologically important species (Allendorf & Hard, 2009; Hendry et al., 2008; Palkovacs et al., 2012). A desire to return traits and their associated ecological functions to historical conditions has led to an increasing interest in evolutionary restoration (Carroll et al., 2014; Hendry et al., 2011; Smith et al., 2014). In this study, we examined the impact of anthropogenic disturbance on the loss of genetic variation associated with anadromous migratory behavior in coastal California steelhead trout. We estimated the impacts of various anthropogenic factors on adaptive genomic variation in a migration‐associated region (MAR) of the *O. mykiss* chromosome 5. Based on the anthropogenic factors associated with the loss of anadromy‐associated alleles, we evaluated the potential for evolutionary restoration at sites across California, USA. Finally, we estimated the financial cost of implementing various proposed restoration efforts across our study watersheds, with the goal of promoting evolutionary restoration of anadromy for the lowest economic cost.

We examined the impacts of climate, geomorphology, and migratory barriers on the frequency of anadromy‐associated alleles. Migratory barriers were found to have the greatest association with anadromous allele frequencies. Natural barriers (e.g., waterfalls, cascades) represent long‐term migratory barriers and had the largest effect, the anadromous allele frequency being on average 31% lower when present. Complete anthropogenic barriers (mostly dams) also had a relatively large effect, with the anadromous allele frequency being an average of 18% lower when complete barriers were present. Most California dams have been operating for <100 years (Hanak et al., 2011); the large effect of complete anthropogenic barriers supports the idea that freshwater residency evolves rapidly following dam construction (Pearse et al., 2014).

While we do not have temporal information from most sites to estimate the rate of allele frequency change following barrier introduction, we can draw some inferences from below–above barrier population comparisons. There are three cases where we have estimates of neutral genetic divergence and variation at the MAR for above‐ and below‐barrier populations (one from Scott Creek and two from the Santa Ynez River). In these cases, changes in allele frequencies at the MAR (49%–76%) are large relative to the extent of genetic divergence at neutral SNP loci (pairwise *F*_ST_ values all <0.01; Clemento et al., 2009; Pearse et al., 2009). Particularly informative is a documented translocation that occurred within the Scott Creek watershed. Here, the frequency of anadromy‐associated MAR alleles is 83% below a barrier waterfall and is reduced to 34% in a population translocated above the waterfall about 100 years ago (*F*_ST_ > 0.3, Martinez, Garza, & Pearse, 2011). These same populations display a pairwise *F*_ST_ of 0.018 at neutral SNP loci (Pearse et al., 2009), clearly demonstrating that drift is not solely responsible for the large‐magnitude directional changes in allele frequencies detected at the MAR. The translocated Scott Creek population above the waterfall currently shows an anadromous allele frequency similar to populations above dams, which were probably isolated for a similar amount of time. Assuming that the below‐barrier population on Scott Creek has not changed dramatically in its allele frequency over the past 100 years, we can infer that the reduction of anadromy‐associated alleles occurred at a rate of approximately 0.05% per year. We anticipate that this rate of change was likely much greater in the years immediately following the translocation and has slowed markedly since then (Kinnison & Hendry, 2001).

Waterfalls, dams, and other impassable barriers are not the only types of migratory barriers found to impact the frequency of anadromy. Partial barriers impart an energetic cost to migration (Hendry et al., 2004; Kendall et al., 2015; Kinnison, Unwin, & Quinn, 2003). When anthropogenic partial barriers were present, anadromous allele frequencies were on average about 2% lower per barrier. While individual partial barriers had a relatively small effect, they occur at very high densities in some watersheds. For example, Boulder Creek (a tributary of the San Lorenzo River in Santa Cruz County; CCC, Fig. S3) has 11 partial anthropogenic barriers, three partial natural barriers, no complete barriers, and an anadromous allele frequency of just 54% (compared to an expected allele frequency of 74% based on its migration distance and number of natural barriers). Thus, the accumulated effects of many partial barriers can have an impact equivalent to that of an impassable dam. Importantly, removal of small partial barriers is less expensive and presents fewer engineering, social, and regulatory challenges compared to large dam removal (Doyle et al., 2005; Graff, 1999).

We found a significant effect of migration distance on the frequency of anadromy‐associated alleles. Migration distance has previously been found to affect anadromy in a wide variety of species, with spawning sites further from the ocean generally displaying lower rates of anadromy (Hendry et al., 2004; Kendall et al., 2015; Ohms et al., 2014). The longer the migration, the more energy must be expended to reach the spawning grounds and the higher the chance of encountering barriers, predators, and other mortality sources. Thus, our results are consistent with prior studies showing that longer migrations select for increased rates of freshwater residency.

In California, steelhead trout are managed in Distinct Population Segments (DPSs) under the US Endangered Species Act. The Southern California DPS had the lowest average anadromous allele frequency measured (Table 3, Fig. S1), most likely due to the high level of human disturbance in Southern California watersheds. A plethora of instream impediments have likely contributed to the overall reduction in the average frequency of anadromy‐associated alleles within its sampled watersheds to just 41%, compared to an expected allele frequency of 62% based on the average migration distance and number of natural barriers. In contrast, the Northern California DPS has the lowest human population, the fewest anthropogenic barriers, and an average frequency of anadromy‐associated alleles of 72% (compared to an expected allele frequency of 73%; Fig. S4). The Central California Coast DPS contains streams that range between highly altered (11–12 partial anthropogenic barriers) to relatively undisturbed (0–2 partial anthropogenic barriers; Fig. S3). While there are no complete anthropogenic barriers in our study populations for the Central California Coast DPS, the accumulation of partial barriers is associated with the reduction in anadromy‐associated alleles in parts of this DPS. The South‐Central California Coast contains the two populations that are outliers in the model (Fig. S3). Both of these populations are in the highly altered Salinas River watershed, which may function differently than other coastal streams due to major anthropogenic disturbances, particularly in the form of intensive agriculture. For example, Tassajera Creek (SCCC, Fig. S2) shows a lower than expected frequency of anadromy‐associated alleles, perhaps due to the agricultural withdrawals that may seasonally dry the Salinas River along much of its main stem channel, creating a low‐flow barrier to migration. In contrast, the Nacimiento River (SCCC, Fig. S2) shows a higher than expected frequency of alleles associated with anadromy. This river flows into the Lake Nacimiento, which may represent the destination for an adfluvial migration, where fish migrate to the lake instead of the ocean (Pearse et al., 2014). Similar adfluvial patterns were found in above reservoir populations around the San Francisco Bay Area (CCC, Fig. S3), where there is a strong association between reservoir size and the frequency of anadromy‐associated alleles (Leitwein et al., 2017).

Each of our study populations were sampled at a single time point, yet we anticipate that allele frequencies at any given site may fluctuate somewhat through time due to drift and dynamic local selective drivers such as stream flow conditions. We do not have the data from repeated sampling events to address within‐site changes in allele frequencies for this study. Nonetheless, our results show that major variation in allele frequencies are predictably related to migration distance and the presence of natural and anthropogenic barriers. These strong and consistent signals would not be expected to emerge in a scenario with high temporal variability in allele frequencies due to random or site‐specific factors. Thus, our overall results are likely robust to fine scale temporal shifts in allele frequencies within sites.

Our study shows that partial and complete anthropogenic barriers are strongly associated with variation in the frequencies of anadromy‐associated alleles. We therefore calculated the expected evolutionary responses for each population in a scenario where all anthropogenic barriers were removed from the downstream watershed. While simulating the removal of large impassable dams yields the biggest predicted evolutionary responses, there are many social, engineering, and legal challenges for projects of this scale (Doyle et al., 2005; Graff, 1999). Smaller dams yield smaller returns, however there are many more of them, which can add up to similar effect contributions to that of a large complete barrier. Smaller scale projects can also be conducted with relative ease by local agencies or watershed stewardship groups. This strategy should be considered as an important complement to large‐scale dam removal when considering the evolutionary restoration of anadromy.

In the subset of watersheds where we evaluated restoration costs, the economic potential of different barrier removal scenarios varied greatly (Table 5). The removal of many smaller partial barriers was substantially cheaper than removing a large impassable dam, yet still achieved a similar evolutionary response. Large dam removals can cost tens of millions of dollars and take decades of planning to complete. For example, the San Clemente Dam Removal Project on the Carmel River in Monterey County, CA, cost approximately $83,000,000 and took 20 years of planning and execution (CalAm 2015). The cost to remediate or remove a small partial barrier on the other hand averaged around $160,000 and some projects can be completed in just under a month (PSMFC, unpublished data). In some locations, complete barriers were poorly designed culverts or flow‐control structures. Removal of these smaller complete barriers could also achieve large gains in anadromy at relatively low costs.

An alternative approach to barrier removal is barrier remediation, which can be conducted on partial and complete barriers. Not all barriers were originally constructed in ways that would allow them to be modified. Nonetheless, some partial barriers such as culverts can be modified to reduce flow velocity and increase water depth, allowing unimpeded passage for anadromous fish. Some dams can have fishways installed, converting them from complete barriers into partial barriers, reducing their impacts substantially. However, fishway construction can be difficult and expensive on some larger dams and many fishways perform poorly, making dam removal the preferred restoration strategy whenever possible (Brown et al., 2013).

Ecologists have called for the use of dam removals as large‐scale experiments to examine ecological processes in rivers and streams (Hart et al., 2002). Our study extends this framework to include evolution. Here we provide predicted evolutionary responses to various restoration scenarios. The next step is to monitor evolutionary change following large‐ and small‐scale barrier removals as management experiments to test these predictions. Evolutionary experiments at this scale are rarely undertaken. Thus, barrier removal provides an important opportunity to achieve restoration objectives while testing basic hypotheses about the factors driving natural selection and evolution in wild populations.

## 5 Conclusions

Human‐induced trait change has been observed in species and ecosystems around the world, and recent efforts have been made to identify and manages these changes (Allendorf & Hard, 2009; Palkovacs et al., 2012). Most evolutionary restoration approaches have focused on manipulating gene flow (Carroll et al., 2014; Hendry et al., 2011; Smith et al., 2014). However, manipulating the environment in ways that shift selection is another method that can effectively restore historical trait values and associated ecological functions (Ashley et al., 2003; Smith et al., 2014). Our study shows that habitat modification in the form of migratory barriers such as dams and culverts are associated with the loss of anadromy‐associated alleles in coastal California steelhead trout populations. While complete barriers such as dams are associated with a dramatic loss of anadromy, the accumulation of large numbers of smaller partial barriers can add up to similarly large impacts. Removing large dams is expected to result in the greatest evolutionary restoration of anadromy, however such projects can be expensive and present many social, engineering, and legal challenges (Doyle et al., 2005; Graff, 1999). Our results suggest that removal of partial barriers can be effective at restoring anadromy at a fraction of the cost. Projects involving small barrier removal present fewer technical and socio‐political challenges. Thus, restoration projects involving the removal of small partial barriers could be considered alongside large dam removals and fishway construction projects as effective tools to restore anadromy to populations that have evolved increased freshwater residency.

## Data Archiving Statement

All data for this study are permanently archived at the Southwest Fisheries Science Center Fisheries Ecology Division (https://swfsc.noaa.gov/FED/).
