# Supplementary material for: Evolutionary restoration potential evaluated through the use of a trait‐linked genetic marker
Source: Evol Appl. 2017 Mar 27;10(5):485–97. doi: 10.1111/eva.12471 (PMC5427673; doi:10.1111/eva.12471)
Supplement: Supplementary file 1 [file EVA-10-485-s001.docx]

**Supporting Information:**

Additional Supporting Information may be found in the online version of this article:

**Figure S1:** The Southern California DPS contains the highest number of complete anthropogenic barriers and the lowest average **f(A)** = 41.02.

**Figure S2:** The South Central California Coast DPS has an average **f(A)** = 71.37. It contains the Salinas River watershed, which has the two outlier populations in our model (Nacimiento River and Tassajera Creek). Tassajera Creek is at the head of a highly agricultural watershed that experiences main stem seasonal drying from agricultural withdrawals. The Nacimiento River population may exhibit adfluvial migrations from Lake Nacimiento.

**Figure S3:** The Central California Coast DPS has the highest average **f(A)** = 75.84. While there are no complete anthropogenic barriers among our sites within this DPS, there are many partial anthropogenic barriers.

**Figure S4:** The Northern California DPS sampling sites have an average **f(A)** = 72.35.

This DPS has no complete anthropogenic barriers and relatively few partial anthropogenic barriers.

**Supplemental Methods 1:** GIS analytical methods for extracting environmental variables for each sampling location.

**Figure S1:** The Southern California DPS contains the highest number of complete anthropogenic barriers and the lowest average **f(A)** = 41.02.

**Figure S2:** The South Central California Coast DPS has an average **f(A)** = 71.37. It contains the Salinas River watershed, which has the two outlier populations in our model (Nacimiento River and Tassajera Creek) perhaps due to main stem seasonally drying from agricultural withdrawals.

**Figure S3:** The Central California Coast DPS has the highest average **f(A)** = 75.84. While there are no complete anthropogenic barriers among our sites within this DPS, there are many partial anthropogenic barriers.

**Figure S4:** The Northern California DPS sampling sites have an average **f(A)** = 72.35.

This DPS has no complete anthropogenic barriers and relatively few partial anthropogenic barriers.

**Supplemental Methods 1:** GIS analytical methods for extracting environmental variables for each sampling location.

Using ArcGIS 10.2 (ESRI 2014), we created a point shapefile for each georeferenced sampling location. We then constructed polyline shapefiles from each respective sampling point to the ocean to represent the stream-path. This was used to calculate Migration Distance because it represents a returning adults migration path. GIS layers for Climatological and Geomorphological variables were found in the OSU Prism, Geospatial Gateway, and CalAtlas databases. We then accessed the *California Fish Passage Assessment Database*, (available at: www.calfish.org/tabid/420/Default.aspx) to identify all the potential barriers to migratory fish along the stream-path present prior to when genetic sampling was performed in 2001. Their classifications are divided up into ‘Complete’, ‘Partial’, ‘Temporal’, and ‘Not a Barrier’. We joined ‘Temporal’ barriers with ‘Partial’ and renamed them *partial* because while they might not be permanent they do impart selective pressure on the populations through time. We further divide them into *natural* and *anthropogenic* to discern between their origins. Examples of temporal barriers are sandbars blocking migration at the mouth of streams as well as flow condition barriers like an impassable cascade at <40 cfs. Partial natural barriers were log jams, small waterfalls, shallow riffles, and temporal barriers. The partial anthropogenic barriers make up the majority of the barriers in this dataset. They consist of diversion dams, fish ladders, flashboard dams, concrete drop structures, weirs, road crossings, and poorly designed culverts. *Complete* barriers represent locations where upstream gene flow was impossible and we further classified both sets of barriers into *natural* and *anthropogenic*. Complete natural barriers consist of waterfalls and inaccessible, steep gradient cascades. Complete anthropogenic barriers were poorly designed culverts and road crossings, grade structures, and dams without fish ladders.

Using the Analysis Toolkit in ArcGIS we conducted a Spatial Join with our Steelhead Sampling Stream polyline layer as the target feature and the four barrier layers as the join features. We used a match option of “WITHIN_A_DISTANCE” to create a buffer of 300ft to capture any barriers whose GPS coordinates were slightly off. Then we removed any extra barriers that were selected at the mouths of tributaries fish did not enter, as they do not block the migration path of the individuals sampled. Conducting this function gave us a count of the number of partial natural and anthropogenic barriers the fish would encounter while migrating to their spawning habitat (or sampling location). It also gave us a count of complete barriers as well, but since they are non-passable, we chose to give them a metric of presence/absence since fish would not be able to encounter more than one. To extract information for Climatological and Geomorphological data we conducted a Spatial Join with the Steelhead Sampling Locations as the target feature and each individual variable as the join features. The match option of “WITHIN_A_DISTANCE” was used to create a 1km buffer to generate a weighted average of the surrounding area. Climatological variables such as rainfall, streamflow, maximum air temperature, etc. were averaged over a five-year period prior to the 2001 sampling year. In this way we could create an average climatological effect that may have affected the sampled populations for the last 1-2 adult generations, which is appropriate given the overlapping generations and iteroparous reproduction of this species. The reason we decided not to use a finer resolution climactic data was that the sheer number of monthly and yearly combinations would have presented logistical difficulties in interpreting the model results.
